# Improvised drainage system for postoperative hematoma in resource-limited settings

**DOI:** 10.1016/j.jdin.2025.09.009

**Published:** 2025-10-03

**Authors:** Mitchell Hanson, Jennifer Easterly, David Harvey

**Affiliations:** a Medical College of Georgia, Augusta, Georgia; b Department of Dermatology, Emory University School of Medicine, Atlanta, Georgia; c Anne Arundel Dermatology, Newnan, Georgia

**Keywords:** dermatologic surgery, health disparities, postoperative hematoma, quality improvement, surgical supplies

## Challenge

Postoperative hematomas are rare in dermatologic surgery but require urgent treatment to prevent ischemia, infection, and flap necrosis.^1^ Standard approaches include aspiration for minor cases or open evacuation with drain placement for larger hematomas. Because hematomas may occur after office hours, prompt recognition and management plans are critical. Many outpatient and rural clinics, particularly in low-resource settings, frequently lack essential supplies due to structural and financial barriers.^2^ Clinicians in such settings often must improvise with available materials to deliver effective, cost-conscious care.

Supplemental MaterialVideoSupplementary Table I, available via Mendeley at https://doi.org/10.17632/xwj98nb39r.1. Video demonstrates surgical drain creation utilizing a https://data.mendeley.com/datasets/rg5kpjsmtk/1 butterfly needle, vacutainer,sterile scissors, #11 blade, hemostat, 4-0 nylon suture, and a large binder clip for securing to the patient’s shirt collar.

## Solution

**Fig 1:**
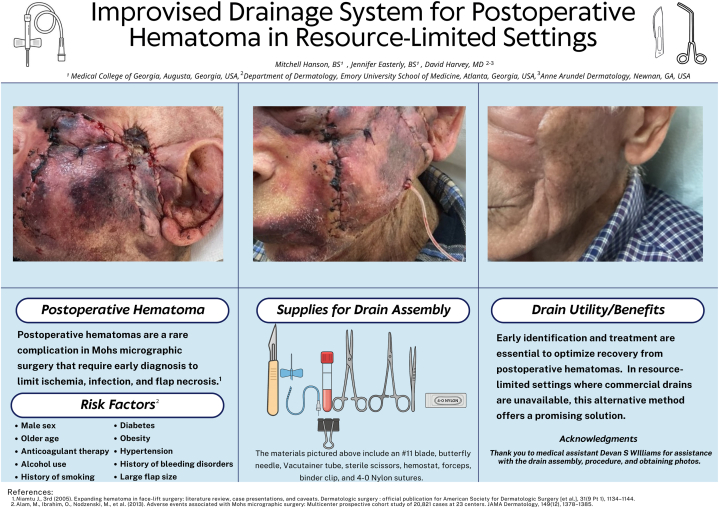
Image of the assembled drain apparatus and tools used.

**Fig 2:**
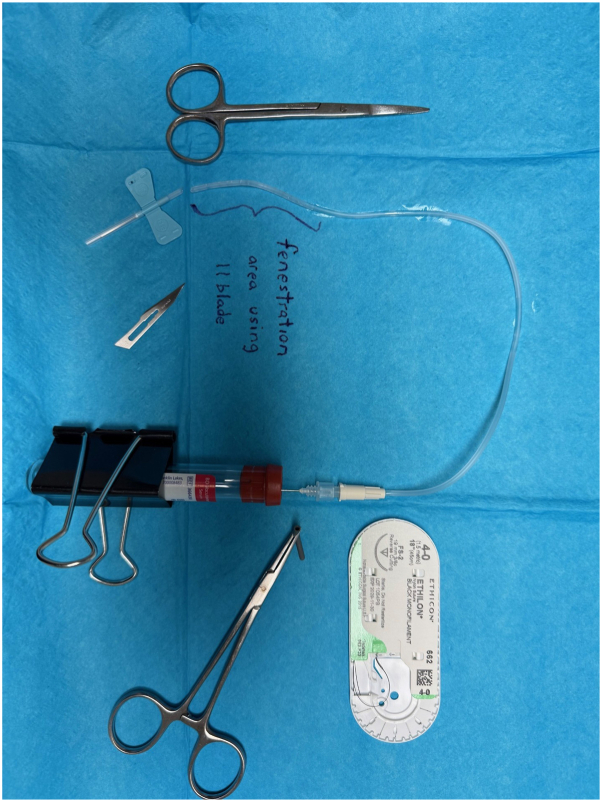
Image demonstarting the region where fenestrations are made and how butterfly attachement is configured for optimal placement.

We present a successful approach to managing a postoperative hematoma in a resource-limited clinic where commercial Jackson-Pratt drains were unavailable. Instead, a closed, negative-pressure drainage system was created using a sterile 23-gauge butterfly needle and a vacutainer tube (Fig 1). The butterfly needle was modified to isolate the tubing apparatus. Using sterile scissors, the tubing was cut at an angle to remove the end containing the sharp butterfly needle. A #11 blade was used to create multiple fenestrations along the distal third of the tubing, allowing for adequate blood flow through the apparatus. The needle connector of the butterfly was inserted, after removal of its rubber sleeve, into the vacutainer’s stopper to create suction (Fig 2). The angulated distal end of the tubing was inserted several centimeters into the hematoma and secured to the skin using a 4-0 Nylon suture. The vacutainer apparatus was secured to the patient’s shirt using a binder clip. Over 5 days, 25 mL of fluid was collected; the drain and sutures were removed after hematoma output diminished.

This improvised method offers a practical, low-cost alternative when standard drains are unavailable, costing approximately $1.50 compared to $11 to $20 for a commercial Jackson-Pratt drain. Limitations include smaller capacity and the need to replace the vacutainer when full or if suction is lost. Although effective in this case for urgent management, further testing on a larger population is indicated to confirm its efficacy as a potential replacement for standard drains. Nonetheless, this approach provides an accessible solution in underserved settings and may prove useful in higher-resource environments during unexpected supply disruptions. As global supply chains remain vulnerable to crises and mismanagement, simple, innovative techniques like this could help ensure continuity of care while minimizing costs and improving patient outcomes.^2^

## Conflicts of interest

None disclosed.

## Figures and Tables

**Still Image:**
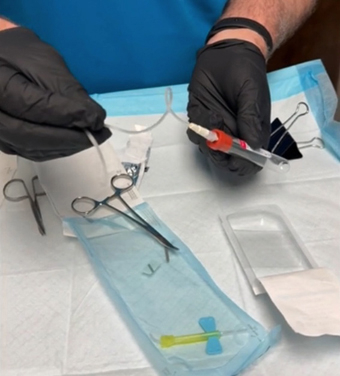


## References

[1] Niamtu J. (2005). Expanding hematoma in face-lift surgery: literature review, case presentations, and caveats. Dermatol Surg.

[2] Meara J.G., Leather A.J., Hagander L. (2016). Global surgery 2030: evidence and solutions for achieving health, welfare, and economic development. Int J Obstet Anesth.

